# A phase I trial of concurrent chemoradiotherapy with non-split administration of docetaxel and cisplatin for dry stage III non-small-cell lung cancer (JCOG9901DI)

**DOI:** 10.1007/s00280-012-1871-5

**Published:** 2012-05-08

**Authors:** Naoya Hida, Hiroaki Okamoto, Yuuki Misumi, Akira Sato, Mari Ishii, Fumihiro Kashizaki, Tsuneo Shimokawa, Teppei Shimizu, Koshiro Watanabe

**Affiliations:** 1 Department of Respiratory Medicine and Medical Oncology, Yokohama Municipal Citizen’s Hospital, 56 Okazawa-cho, Hodogaya-Ku, Yokohama, Kanagawa 240-8555 Japan; 2 Department of Radiology, Yokohama Municipal Citizen’s Hospital, 56 Okazawa-cho, Hodogaya-Ku, Yokohama, Kanagawa 240-8555 Japan

**Keywords:** Non-small-cell lung cancer, Locally advanced, Concurrent chemoradiation, Cisplatin, Docetaxel

## Abstract

**Purpose:**

This study aimed to establish the maximum tolerated dose of concurrent chemoradiotherapy (cCRT) with conventional administration of the docetaxel (D) plus cisplatin (P) (conv-DP) regimen.

**Methods:**

Patients (aged ≤70 years) with unresectable dry stage III non-small-cell lung cancer (NSCLC) and having performance status 0 or 1 and adequate organ function were eligible. They received radiotherapy (60 Gy in 30 fractions) once daily starting on day 2. Concurrent P (day 1; 60 mg/m^2^ at Levels 1–3, 80 mg/m^2^ at Level 4) and D (day 1; 30 mg/m^2^ at Level 1, 40 mg/m^2^ at Level 2, 50 mg/m^2^ at Levels 3–4) were administered every 4 weeks for 2–4 courses.

**Results:**

Eighteen patients were enrolled (stage IIIA/IIIB, 5/13 patients). Three cases of dose-limiting toxicity were observed in this study, although another 3 cases were added at Levels 2 and 3. Radiotherapy was completed in 15 patients. Seventeen patients received more than 2 courses of chemotherapy. Neither Grade 3/4 esophagitis nor severe hematological events were observed at Levels 1–4. However, dose escalation to Level 5 (P [80 mg/m^2^], D [60 mg/m^2^]) was stopped because the Level 5 dose was the recommended dose (RD) of chemotherapy alone for stage IIIB/IV NSCLC in Japan. Therefore, the RD was determined as D50/P80 mg/m^2^ in this cCRT. The objective response rate was 89 %, and the median survival time was 23.6 months.

**Conclusions:**

cCRT with non-split DP was a tolerable and effective regimen, and RD was 50/80 mg/m^2^ every 4 weeks.

## Introduction

Approximately 30 % of patients with non-small-cell lung cancer have unresectable locally advanced disease (LA-NSCLC) at diagnosis. Before the 1980s, thoracic radiotherapy (TRT) was the standard treatment for these patients. In the 1980s, several studies demonstrated that 2 cycles of chemotherapy followed by radiation improved the median survival time by approximately 3 months and 5-year survival by 3–10 % compared with TRT alone [1, 2]. In the 1990s, studies from the United States [3], Japan [4], and elsewhere demonstrated that concurrent administration of 2 cycles of chemotherapy with TRT improved the median survival time by additional 3 months and 5-year survival by an additional 5 % compared with sequential chemoradiotherapy. Therefore, treatment recommended for LA-NSCLC patients who have a good performance status (PS) is concurrent chemoradiotherapy (cCRT). In the 1990s, platinum-based third-generation chemotherapy (i.e., paclitaxel, vinorelbine, and docetaxel) was shown to be superior to second-generation chemotherapy (i.e., etoposide, vindesine, and mitomycin) in treating metastatic NSCLC [5–7]. However, full-dose chemotherapy with cCRT using a platinum-based third-generation doublet is considered to have unacceptable toxicity. Therefore, for both reduction in toxicity and enhancement of the radiosensitizing effect, weekly split chemotherapy has often been used in chemoradiotherapy with a platinum-based third-generation doublet. In the curative setting, distant metastasis control is one of the most important factors. Furuse et al. [4] reported that the distant relapse rate was 64 % among patients treated with mitomycin, vindesine, and cisplatin as cCRT. To prevent distant metastatic relapse, it is necessary to enhance the effect of chemotherapy. In metastatic NSCLC, chemotherapy with cisplatin and docetaxel (DP) is one of the most effective regimens [7]. Thus, to maximize chemotherapeutic effectiveness, we used DP as concurrent chemotherapy in both conventional and non-split administration. This phase I study aimed to establish the maximum tolerated dose (MTD) of chemoradiotherapy with the conventional administration of DP therapy.

## Patients and methods

### Eligibility criteria

Staging for enrollment criteria was performed according to the lung cancer staging system of the International Union against Cancer [8]. Staging procedures included chest radiograph, computed tomography (CT) scan of the chest, CT scan or magnetic resonance imaging (MRI) of the brain, CT scan or ultrasound of the abdomen, and isotope bone scanning. Lymph nodal involvement was mainly based on size criteria indicated in the chest CT scan. The mediastinal lymph node beyond 10 mm in the short axis diameter was considered as involvement node. Patients with histologically or cytologically documented LA-NSCLC were enrolled in this study. Other eligibility criteria included the following: (1) unresectable clinical stage IIIA/IIIB on examination 2 weeks before enrollment; (2) age ≤70 years; (3) Eastern Cooperative Oncology Group (ECOG) PS 0 or 1; (4) measurable or assessable tumors; (5) adequate bone marrow function (white blood cell count ≥4,000/mL and ≤12,000, platelet count ≥10 × 10^4^/mL, and hemoglobin level ≥10 g/dL), renal function (serum creatinine (Cr) level ≤1.5 mg/dL or creatinine clearance (Ccr) ≥60 mL/min), hepatic function (bilirubin level ≤1.5 times upper limit of normal and aspartate aminotransferase (AST) ≤2 times upper limit of normal), and pulmonary function (arterial blood oxygen (PaO_2_) ≥70 mmHg); (6) life expectancy >8 weeks; (7) predicted area of the radiation field was less than half of 1 lung; (8) absence of previous chemotherapy or TRT; and (9) no previous or concurrent malignancy. Exclusion criteria included interstitial pneumonitis or pulmonary fibrosis, pleural or pericardial effusion, severe superior vena cava syndrome requiring emergent radiotherapy, active infection, poorly controlled diabetes mellitus, uncontrollable cardiac arrhythmia or hypertension, and acute myocardial infarction within 3 months before study enrollment. All patients gave written informed consent according to institutional guidelines. This protocol was approved by the Ethical Committee of Japan Clinical Oncology Group (JCOG) and our institute.

### Treatment plan

In every case, CT results were used to guide radiotherapy. Moreover, the more accurate three-dimensional conformal radiotherapy (3D-CT) simulation technique was used in 12 of 18 cases since May 2001 in our institution. The initial 6 cases were radiated by the conventional radiation method but not by the 3D technique. Radiotherapy was administered using an angled field technique modulated on the volume and location of the disease so as to include 100 % of the target volume in the isodose, with a maximum dose to the spine of 50 Gy.

The initial opposing anterior–posterior treatment fields encompassed the primary tumor, bilateral mediastinal lymph nodes, and ipsilateral hilar nodes. The supraclavicular nodes were included within the field in case of availability of clinical evidence of their involvement. The gross tumor volume was the clinical target volume (CTV), and the planning target volume was CTV plus the surrounding 1.5-cm margin. The total referred dose was 60 Gy with a classical fractionation of 2 Gy/day (consecutive 5 days/week). Concurrent radiotherapy began on the day after chemotherapy started (day 2). The maximum duration of radiotherapy was 55 days. Lung parenchyma correctional factors and linear accelerator with photon regimen (nominal energy 6–10 MV) were used in all cases.

On days 1 and 29, docetaxel was intravenously administered for 1 h followed by a 2-h infusion of cisplatin. Concurrent treatment with antiemetics, hydration, antibiotics, sedatives, cortisone, and gastric protectors was permitted. Up to 2 courses of consolidation chemotherapy with the same regimen (cisplatin and docetaxel every 28 days) was permitted after 2 courses of cCRT.

During chemoradiation, if the white blood cell (WBC) count was <1,000/mm^3^ or absolute neutrophil count (ANC) was <500/mm^3^, radiotherapy was stopped and daily granulocyte colony-stimulating factor (G-CSF) was subcutaneously administered until the WBC count increased to 2,000/mm^3^. If the platelet count was <5 × 10^4^/mm^3^ or PaO_2_ decreased by ≥10 mmHg from baseline, radiotherapy was stopped. If the WBC count was >2,000/mm^3^, ANC count was >1,000/mm^3^, platelet count was >5 × 10^4^/mm^3^, or PaO_2_ was decreased by <10 mmHg from baseline, radiotherapy was restarted. Radiotherapy and concomitant use of G-CSF was contraindicated. If esophagitis of Grade 3 or higher occurred, radiotherapy was stopped until recovery to Grade 2 or lower. If hematological toxicities rated as Grade 4 occurred during the first course of chemotherapy, the dose of docetaxel was reduced by 25 %.

When the second course of chemotherapy was started, each patient was required to meet the following criteria: WBC count >3,000/mm^3^, platelet count >7.5 × 10^4^/mm^3^, AST and/or alanine aminotransferase (ALT) ≤2.5 × normal upper limit, PS 0–2, and Cr level ≤1.5 mg/dL. If the aforementioned criteria were not met, only radiotherapy was started. When the criteria were met, chemotherapy was started as soon as possible. If the second course was delayed 2 weeks or more because of toxicity, further chemotherapy was discontinued and only radiotherapy was used. If Cr was ≤1.5 mg/dL and Ccr was ≥60 mL/min on the day chemotherapy was started, the full dose of cisplatin was administered. If Cr was ≤1.5 mg/dL and Ccr was within the range of 40–60 mL/min, a 75 % dose of cisplatin was administered. If Ccr was <40 mL/min, chemotherapy was stopped. During chemotherapy alone, the dose modification schedule was almost the same as that during the chemoradiation period. If the same toxicities were observed after dose reduction, the protocol treatment was terminated.

### Treatment evaluation

Tumor response and toxicity were evaluated according to World Health Organization response criteria [9] and Japan Clinical Oncology Group (JCOG) toxicity criteria [10], respectively. Extramural review was not performed. During the treatment, complete blood cell count and routine blood chemistry were examined two times a week and PaO_2_ and chest radiographs were examined at least once a week until the patient had apparently recovered from all acute toxic effects. Dose-limiting toxicity (DLT) was evaluated by administering 2 courses of chemotherapy to each patient. The objective response rate (ORR) was defined as the proportion of patients (out of all eligible patients) with complete response (CR) or partial response (PR). Overall survival (OS) was measured from the date of patient registration to the date of death from any cause. If a patient was alive at the final follow-up survey, OS was censored at the last contact date. The estimates of survival distribution were calculated using the Kaplan–Meier method.

### Study design

**Fig. 1:**
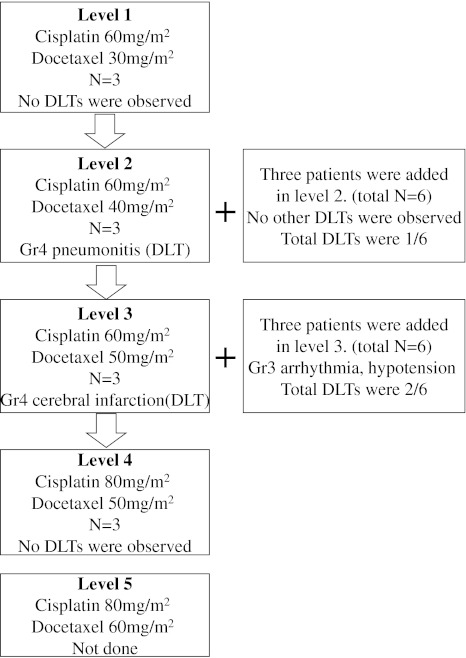
Dose escalation and study procedure

This study was a phase I dose escalation study conducted at a single institution (Yokohama Municipal Citizens Hospital, Yokohama, Japan) and was designed to define MTD of both cisplatin and docetaxel when combined with concurrent TRT. The first dose level consisted of cisplatin 60 mg/m^2^ and docetaxel 30 mg/m^2^. The dose escalation plan and study procedure is shown in Fig. 1. Dose-limiting toxicity was defined as Grade 3 or Grade 4 non-hematological toxicity excluding nausea or vomiting and alopecia, Grade 4 neutropenia lasting 4 days or more, Grade 4 febrile neutropenia, Grade 4 thrombocytopenia, Grade 3 or higher esophagitis, or acute interstitial pneumonia (any grade) during 2 courses of chemotherapy. Patients who could not meet the criteria for the next course of chemotherapy after more than 6 weeks had passed from the time of the last treatment were considered to have developed DLT. If 1 or 2 instances of DLT were observed among 3 patients, 3 additional patients were to be treated at the same dose level. Dose escalation continued if DLT was observed in no more than 3 of 6 patients. If 3 of 3 patients or at least 4 of 6 patients showed DLT at a given dose level, then that level was considered to be MTD and 1 dose level below that level to be RD.

## Results

### Patient characteristics

Between July 1999 and May 2006, 18 patients were enrolled in this trial. Median age was 60 years (range, 43–70 years) and PS of 1 was observed in 14 patients. Clinical staging identified 5 patients as stage IIIA and 13 patients as stage IIIB. Histology was confirmed as follows: adenocarcinoma in 9 patients (50 %), squamous cell carcinoma in 7 patients (39 %), and large cell carcinoma in 2 patients (11 %) (Table 1).

**Table 1:** Patient characteristics

No. of patients	18
Gender	
Male	13
Female	5
Age	
Median (Range)	60 (43–70)
Histology	
Squamous cell carcinoma	7
Adenocarcinoma	9
Large cell carcinoma	2
Stage	
IIIA	5
IIIB	13
ECOG-PS	
0	4
1	14

*ECOG,* eastern cooperative oncology group; *PS,* performance status

### Dose escalation procedure

The procedures followed in this phase I study are shown in Fig. 1. No DLTs were observed at Level 1. At Level 2 (cisplatin 60 mg/m^2^ and docetaxel 40 mg/m^2^), 1 of 6 patients showed DLT. The patient was a 67-year-old man who was an ex-smoker (100 packs/year). The comorbidity of this patient was cardiac dysfunction due to mitral valve regurgitation. Hypoxia and interstitial shadow developed at day 29 after the initiation of the protocol treatment. Despite steroid pulse therapy following immediate discontinuation of chemoradiotherapy, this patient died of respiratory failure and progression of lung cancer on day 83. This case was considered to be Grade 4 pneumonitis (unrecovered). Although additional 3 patients were added at Level 2, no other DLTs were observed; thus, the dose was escalated to Level 3 (cisplatin 60 mg/m^2^ and docetaxel 50 mg/m^2^). However, one of the first 3 patients added at Level 3 had DLT. This 58-year-old woman developed Grade 4 cerebral infarction. The patient developed right hemiparesis and aphasia at 3 weeks after the first course of chemotherapy and 30 Gy of irradiation. She recovered from hemiparesis but mild aphasia remained. After the patient recovered from the severe toxicity, thoracic irradiation was continued up to 60 Gy. We considered this event to be a Grade 4 adverse event and judged it to represent DLT. Although additional 3 patients were added at Level 3, 1 patient developed Grade 3 atrial fibrillation and hypotension 5 days after the second course of chemotherapy. This patient completely recovered without complications within a few days. Because only 2 of 6 patients had DLTs at Level 3, the dose was escalated to Level 4 (cisplatin 80 mg/m^2^ and docetaxel 50 mg/m^2^); at this level, no DLTs were encountered. However, dose escalation to Level 5 (cisplatin 80 mg/m^2^ and docetaxel 60 mg/m^2^) was stopped because the Level 5 dose was the recommended dose of chemotherapy alone for stage IIIB/IV NSCLC in Japan. This decision was approved by the JCOG Data and Safety Monitoring Committee. Thus, although MTD was not obtained in this study, the recommended dose was considered to be cisplatin 80 mg/m^2^ and docetaxel 50 mg/m^2^.

Seventeen patients received more than 2 courses of chemotherapy. Median interval by third course was 28 days, and no dose reductions were observed in all courses. Due to disease progression, one patient received only one course of chemotherapy. Eight patients received additional 2 courses of consolidation chemotherapy (Table 2). Radiotherapy (60 Gy) was completed in 15 patients (Table 3).

**Table 2:** Chemotherapy interval and dose reduction

Chemotherapy course	*N*	Days	Dose reduction
1–2 course	17	27–35 (median 28)	–
2–3 course	9	28–32 (median 28)	0
3–4 course	8	28–38 (median 29)	0

**Table 3:** Radiotherapy delivery

Level	*N*	RT dose (Gy)	With RT delay
1	3	60, 60, 60	1 (3 days)
2	6	60, 36^a^, 60	0
		60, 60, 56^b^	
3	6	60, 60^c^, 60	
		60, 60, 60^d^	2
4	3	60, 60, 60	0

*No/N,* number; *RT,* radiation therapy; *DLT,* dose-limiting toxicity

^a^Died with Gr4 pneumonitis 58 days after 2nd course of chemotherapy (DLT)

^b^Gr2 infection

^c^Gr4 cerebral infarction

^d^Gr3 Atrial fibrillation and hypotension

### Toxicities

Therapeutic toxicities are summarized in Table 4. Grade 3 leukocytopenia, neutrocytopenia, and thrombocytopenia were observed in 5 patients (28 %), 4 patients (22 %), and 1 patient (6 %), respectively. No Grade 4 hematological event was observed. No Grade 3 or higher esophagitis or other gastrointestinal toxicities were observed. Other severe (Grade 3 and higher) toxicities were determined to be DLTs and are described above. In addition, no late toxicities (i.e., radiation pneumonitis, prolonged esophagitis, or spinal cord toxicities) were observed in all long-term survivors.

**Table 4:** Toxicities (worst of any course)

Level	Hematological toxicities							Hb		PLT			Gastrointestinal toxicities			
	*N*	WBC			Grade ANC								N and V	Grade diarrhea	Esophagitis	
		2	3	4	2	3	4	2	3	2	3	4	≥2	≥2	2	3 or 4
1	3	1	0	0	1	0	0	2	0	0	0	0	0	0	0	0
2	6	2	1	0	5	1	0	1	0	0	0	0	0	0	1	0
3	6	2	3	0	3	2	0	3	0	0	1	0	2	1	1	0
4	3	0	1	0	0	1	0	0	0	0	0	0	2	0	2	0

*N,* number; *WBC,* white blood cell; *ANC,* absolute neutrophil count; *Hb,* Hemoglobin; *PLT,* platelet, N and V, nausea and vomitting

### Responses, recurrence pattern, and survival

All 18 patients enrolled were considered for response on an intent-to-treat basis. Overall, 16 patients showed an objective response to treatment (no CRs), yielding an 89 % response rate. The pattern of initial recurrence is shown in Table 5. Distant relapse (67 %) was higher than locoregional relapse (33 %). At the time of this report, 14 deaths had occurred. The median follow-up for overall and surviving patients was 19.8 (range, 2.8–69.7) and 42.9 (range, 19.9–69.7) months, respectively. The median progression-free survival was 8.4 months. The median survival time was 23.6 months, with an estimated 2-year survival rate of 43 %.

**Table 5:** Pattern of initial recurrence (*N* = 12)

Recurrence site	No. of patients (%)
Locoregional only	4^a^ (33)
Distant	8 (67)
Brain	2 (17)
Other	6^b^ (50)

^a^One pleural effusion included

^b^Metastatic site: supraclavicular lymphnode (1 patient), bone (1 patient), liver (1 patient), and pulmonary metastasis (3 patients)

## Discussion

We evaluated the use of simultaneous irradiation with coadministration of a third-generation agent doublet to enhance the effect of chemotherapy. Specifically, our phase I study explored the safety and optimal dose of conventional and non-split administration of cisplatin and docetaxel therapy as cCRT. The optimal regimen, dosage, and administration of a third agent for LA-NSCLC are controversial. Combination chemotherapy using a reduced or fractionated dose of platinum plus third-generation agents has been administered to reduce toxicity in many clinical trials [11, 12]. However, in our study, the chemotherapy dose was escalated to almost the recommended full dose of chemotherapy administered alone for metastatic NSCLC in Japan. The dose was escalated to cisplatin 80 mg/m^2^ and docetaxel 50 mg/m^2^ (Level 4), which is close to the full dose. The protocol specified further escalation to the recommended full dose of cisplatin 80 mg/m^2^ and docetaxel 60 mg/m^2^ (Level 5). However, no new anticancer drug has been used concomitantly with cisplatin-based chemotherapy and radiotherapy at a full dose, and Level 5 was the recommended dose of chemotherapy alone for stage IIIB/IV NSCLC in Japan. Thus, chemotherapy at Level 5 was not conducted, as agreed by the JCOG Data and Safety Monitoring Committee. Therefore, a dose of cisplatin 80 mg/m^2^ and docetaxel 50 mg/m^2^ (Level 4) is recommended for future study.

A treatment strategy with chemoradiotherapy aimed at a complete cure of LA-NSCLC should include both local and distant disease control. Local control improves by simultaneous radiotherapy. Docetaxel enhances the cytotoxic effects of radiotherapy in vitro [13, 14], with radiation enhancement being superior to that observed with paclitaxel [15]. The combined administration of cisplatin and irradiation improved survival and decreased the local failure rate, although the addition of relatively low doses of cisplatin did not decrease the distant failure rate [16–18].

To enhance local tumor control without increasing toxicity, we could use the 3D-CRT technique in the study. Although there are only a few small studies of radiation dose escalation using 3D-CRT [19–21], this technique is expected to reduce radiation-related toxicity. Large-scale trials are necessary to evaluate whether better tumor control because of the higher doses and reduced toxicity is associated with this technique. This outcome might be expected because higher doses of radiation can be delivered to the tumor while decreasing the dose of radiation administered to surrounding healthy tissues.

Distant disease control mainly depends on the strength of chemotherapy. In chemoradiotherapy, divided doses of many third-generation anticancer drugs have been used to reduce toxicity. Consequently, although toxicity is clearly reduced, the antitumor effect throughout the entire body may be decreased. Docetaxel is one of the most effective antitumor agents; therefore, we expected that conventional and non-split administration of docetaxel would provide improved efficacy for the entire body than split administration. However, of the 12 patients who had disease relapse in this study, the initial site of recurrence was distant in 8 patients and local in 4 patients. These results were unexpected; however, patients treated with low-dose chemotherapy (Levels 1–3) were included in this group. Further studies are required to investigate this issue.

Prophylactic cranial irradiation (PCI) in LA-NSCLC has been discussed because the brain is the first site of distant recurrence in many treated patients. In a comparative study of additional PCI after chemoradiotherapy in LA-NSCLC patients, Gore et al. and Sum et al. found that PCI affected the time to brain metastasis and quality of life but did not improve survival [22, 23]. Dimitropoulos et al. [24] reported the maximum benefit of PCI may bestow on younger smokers, which is not mainly population of NSCLC. The usefulness of PCI in LA-NSCLC remains controversial. In our study, 8 of 18 patients were treated with consolidation chemotherapy, although there has been no definitive evidence for using consolidation chemotherapy after chemoradiation therapy. Additional consolidations consisting of docetaxel [25] or gefitinib [26] after the induction of cisplatin plus etoposide reported no effect on survival. Although consolidation chemotherapy may have a possible effect in reducing distance metastasis, its role after induction chemoradiotherapy remains controversial [27].

Grade 3 or higher non-hematological toxicity occurred in 3 patients. One patient had Grade 4 pneumonitis during chemoradiotherapy at Level 2. This patient had cardiac dysfunction, and the irradiation field was relatively extensive, although covering less than half of 1 lung. No other cases of serious pneumonitis were observed. The other 2 adverse events were cerebral infarction associated with mild paralysis and transient atrial fibrillation associated with hypotension. However, these adverse events were incidental. Overall toxicity was generally mild; specifically, no Grade 3 or higher esophagitis was found.

It is difficult to evaluate efficacy because this study was conducted as a phase I dose escalation trial. However, the overall response rate (PR + CR) was 89 %, median progression-free survival was 8.4 months, median survival time was 23.6 months, and the 2-year survival rate was 43 %, that is, the overall outcomes were promising compared with the results of recent randomized phase III trials. Studies OSCLG0007 [11] and WJTOG0105 [12] were randomized controlled trials of cCRT for LA-NSCLC using platinum and a third-generation doublet. These studies reported that the median OS of the experimental arm was 19.8–26.8 months. Ohyanagi et al. [28] reported the results of a phase II trial of cisplatin (60 mg/m^2^, day 1) and S-1 (orally at 40 mg/m^2^ daily, days 1–14) administered as conventional (non-fractionated) chemotherapy with concurrent radiotherapy. These authors reported an excellent median survival of 33.1 months and a distant failure rate of 50 %. Based on our promising phase I trial, a randomized comparative study of cisplatin plus docetaxel or TS-1 with concurrent radiotherapy is being conducted by the Thoracic Research Oncology Group (TORG) of Japan.

In conclusion, cCRT with non-split DP therapy seemed to be a tolerable and effective regimen for NSCLC patients in our phase I study. RD for DP was 50 and 80 mg/m^2^ every 4 weeks. The use of cCRT with near full dose, non-split administration of cisplatin and a third-generation drug appears to be a promising strategy. A further trial is being planned to evaluate the efficacy and toxicity of this multimodal therapy.
